# An integrated culturomic and genomic database and analysis platform for methanogenic archaea

**DOI:** 10.1093/database/baag055

**Published:** 2026-09-15

**Authors:** Jiuyu Chen, Shijie Ren, Zhenye Tong, Zhaofeng Guan, Wenhao Zhang, Siwei Ren, Liqian Ma, Lingdou Kong, Hengyao Chong, Zihao Wang, Xiaoyu Yong, Su Yan, Yuanpeng Wang, Jun Zhou

**Affiliations:** Bioenergy Research Institute, College of Biotechnology and Pharmaceutical Engineering, Nanjing Tech University, Nanjing, Jiangsu 211816, China; Bioenergy Research Institute, College of Biotechnology and Pharmaceutical Engineering, Nanjing Tech University, Nanjing, Jiangsu 211816, China; Bioenergy Research Institute, College of Biotechnology and Pharmaceutical Engineering, Nanjing Tech University, Nanjing, Jiangsu 211816, China; Bioenergy Research Institute, College of Biotechnology and Pharmaceutical Engineering, Nanjing Tech University, Nanjing, Jiangsu 211816, China; Bioenergy Research Institute, College of Biotechnology and Pharmaceutical Engineering, Nanjing Tech University, Nanjing, Jiangsu 211816, China; Bioenergy Research Institute, College of Biotechnology and Pharmaceutical Engineering, Nanjing Tech University, Nanjing, Jiangsu 211816, China; Bioenergy Research Institute, College of Biotechnology and Pharmaceutical Engineering, Nanjing Tech University, Nanjing, Jiangsu 211816, China; Bioenergy Research Institute, College of Biotechnology and Pharmaceutical Engineering, Nanjing Tech University, Nanjing, Jiangsu 211816, China; Bioenergy Research Institute, College of Biotechnology and Pharmaceutical Engineering, Nanjing Tech University, Nanjing, Jiangsu 211816, China; Bioenergy Research Institute, College of Biotechnology and Pharmaceutical Engineering, Nanjing Tech University, Nanjing, Jiangsu 211816, China; Bioenergy Research Institute, College of Biotechnology and Pharmaceutical Engineering, Nanjing Tech University, Nanjing, Jiangsu 211816, China; Bioenergy Research Institute, College of Biotechnology and Pharmaceutical Engineering, Nanjing Tech University, Nanjing, Jiangsu 211816, China; Department of Chemical and Biochemical Engineering, College of Chemistry and Chemical Engineering, Key Laboratory for Chemical Biology of Fujian Province, Xiamen University, Xiamen, Fujian 361005, China; Bioenergy Research Institute, College of Biotechnology and Pharmaceutical Engineering, Nanjing Tech University, Nanjing, Jiangsu 211816, China

## Abstract

Methanogenic archaea research is challenged by limited strain resources, fragmented genomic data, inconsistent genome quality, substantial uncultured lineages, and difficulties in laboratory culturing, hindering advances in biogas production, climate mitigation, and microbial ecology. These archaea play crucial roles in global carbon cycling and anaerobic environments, yet scattered data and unculturable strains limit systematic studies and applications. To address this, we created MethArDB (Methanogenic Archaeal Genome Database), a specialized database for methanogenic archaea, compiling 3919 genomes, 87 host-associated plasmids, and 42 phages, with standardized quality classifications (complete, scaffold, draft), protein sequences, and metadata on geography, habitats, metabolism, and inheritable elements. Integrated MethArCT (Methanogenic Archaeal Culturomics Toolkit) employs a dual-threshold orthologous/paralogous protein analysis to evaluate metabolic pathway completeness, predicting cultivation parameters and suggesting candidate cultivation strategies, including potential medium formulations and conditions, to support strain isolation. Overall, MethArDB and MethArCT form an integrated platform combining genomics and culturomics to facilitate methanogenic archaea research.

**Database URL:**  http://methardb.cn

## Introduction

Methanogenic archaea are obligate anaerobes that play a pivotal role in the global carbon cycle as the dominant producers of atmospheric methane [1]. They are also of critical importance for carbon neutrality strategies, bioenergy production, and the study of life’s origins [2–4]. However, despite the successful cultivation of many methanogenic archaea, numerous lineages particularly those identified through metagenomic studies and representing ‘microbial dark matter’ [5] remain uncultured, reflecting ongoing challenges in their isolation and cultivation.

In recent years, rapid advances in metagenomics and single-cell sequencing technologies have led to a dramatic increase in the availability of genomic data for methanogenic archaea [6], providing unprecedented opportunities to investigate their genetic basis and physiological functions. However, the fragmentation of genomic resources and analytical tools still severely hinders research progress. Although databases such as NCBI, Genome Taxonomy Database (GTDB), and Kyoto Encyclopedia of Genes and Genomes (KEGG) hold a large number of methanogenic archaeal genomes, these data lack targeted systematic integration. They also lack a unified and robust quality control mechanism, with high-quality complete genomes and well-assembled scaffolds often mixed with low-quality contigs and potentially contaminated metagenome-assembled genomes (MAGs). In addition, researchers usually need to employ a suite of tools, such as CheckM2 for quality assessment, RagTag or Ragout for assembly optimization, Methane Cycling Database (MCycDB) or KEGG for metabolic pathway prediction, and IQ-TREE or EzAAI for phylogenetic analysis [7–13]. Such fragmentation results in cumbersome, inefficient, and non-standardized analytical workflows.

Furthermore, the prediction of methanogenic metabolic pathways is impeded by the fact that paralogous proteins exhibit high structural similarity but distinct functions, which can lead to the misclassification of metabolic pathways in methanogenic archaea when traditional methods are used. For instance, some obligate hydrogenotrophic methanogens encode both F_420_-reducing hydrogenases and formate dehydrogenases (FdhAB), enabling them to utilize formate as an alternative electron donor. However, accurate identification of such metabolic capabilities can be complicated by the presence of paralogous proteins with high sequence similarity. For example, the hydrogenase subunits ElpAB share sequence homology with FdhAB due to their common evolutionary origin, which may lead to functional misannotation when relying solely on sequence similarity. Despite this similarity, these proteins are functionally distinct and non-interchangeable [14]. Similarly, in hydrogen-dependent methylotrophic methanogenesis, several methyltransferases—including MtsA (methanethiol), MtbA (methylamine), and MtaA (methanol)—share substantial sequence similarity due to their common evolutionary origin, yet exhibit strict substrate specificity. This high similarity among paralogous proteins can lead to misannotation in homology-based analyses, thereby affecting the accuracy of metabolic pathway reconstruction. For example, *Methanofastidiosum* strains encoding MtsA are unable to utilize methylamine or methanol [15]. A similar issue is observed for the alkane-coenzyme M reductase complex AcrABG and the canonical methyl-coenzyme M reductase McrABG, which also exhibit high sequence similarity but catalyse distinct reactions, further highlighting the need for more stringent strategies to distinguish paralogous proteins in functional annotation [16].

Despite advances in methods for deciphering microbial physiological and biochemical traits using genomic information and the increasing availability of extensive strain-level physiological data for bacteria and archaea in databases such as BacDive [17], critical gaps remain. For example, the lack of dedicated bioinformatics tools or databases hinders predictions of cultivation conditions for specific functional groups of microorganisms (e.g. methanogenic, sulphur-metabolizing, and nitrogen-metabolizing microorganisms). This deficiency is particularly pronounced for methanogenic archaea, which typically possess multiple key physiological characteristics, including salinity preference, substrate specificity, and dependence on symbiotic relationships (as evidenced by difficulties in achieving pure culture and frequent observations of abundant associated bacteria via fluorescence microscopy). Furthermore, several methanogenic lineages demonstrate halophilic characteristics [18–22], alongside the capacity for nitrogen [23–25] and sulphur metabolism [26–28]. Currently, the lack of accurate predictive information regarding these key traits, especially salinity preference, substrate specificity, and symbiotic dependence, constitutes a major constraint for the isolation, purification, and high-density enrichment of methanogenic archaea.

**Figure 1:**
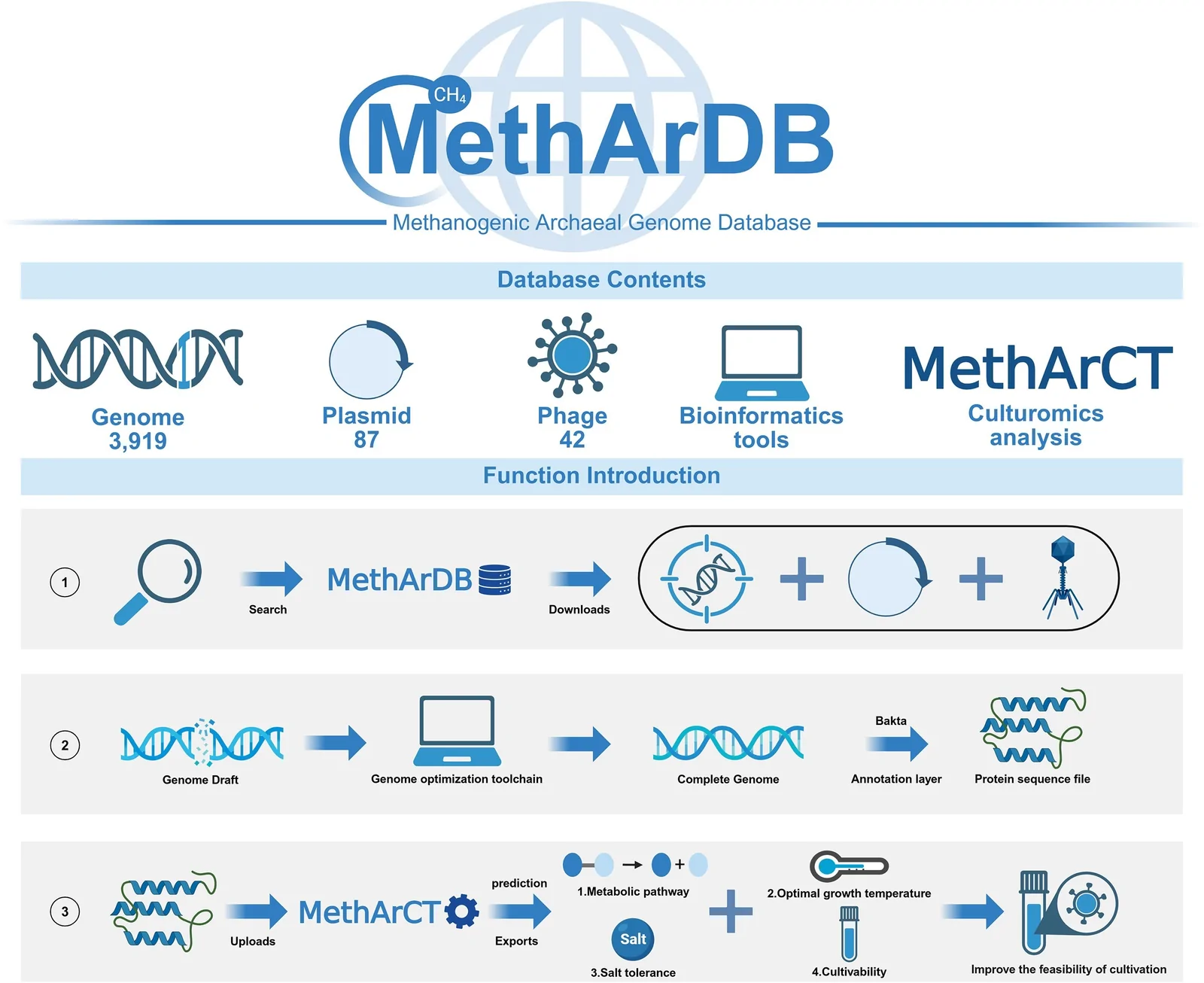
Overview of the MethArDB database and integrated analysis platform. MethArDB integrates publicly available methanogenic archaeal genomes together with associated plasmid and phage datasets and provides a web-based platform for data retrieval and analysis. Users can search and download database records, optimize draft genome assemblies, annotate genomes to generate protein sequence files, and perform downstream analyses using MethArCT. The toolkit supports prediction of methanogenic pathways, optimal growth temperature, salinity tolerance, and predicted culturability.

To address the challenges outlined above, we developed MethArDB (Methanogenic Archaeal Genome Database), the first integrated platform consolidating methanogenic archaeal genomes, associated plasmids, phage sequences, and functional prediction tools. The current release of MethArDB comprises 3919 high-quality genomes, 87 host-associated plasmids, and 42 archaeal phage sequences and is supplemented by an interactive world map that visualizes their geographic and environmental distributions (Fig. 1). All complete genomes are annotated with standardized environmental and metabolic metadata to facilitate comparative cross-strain analyses. The core bioinformatic module, MethArCT (Methanogenic Archaeal Culturomics Toolkit), employs a dual-threshold alignment strategy specifically designed to unambiguously discriminate between orthologs and paralogs. This enables accurate calculation of metabolic pathway completeness based on genomic protein sequences and supports precise prediction of metabolic types. Furthermore, MethArCT incorporates machine learning and algorithmic approaches to predict optimal physiological conditions and integrates quality assessment tools such as CheckM2 [13], thereby providing a theoretical basis for guiding targeted isolation and cultivation strategies of uncultured lineages. By consolidating previously fragmented genomic resources and enabling accurate annotation and prediction of culturability, MethArDB establishes a unified research framework and a robust technical foundation to address two long-standing bottlenecks in methanogen research: their recalcitrance to cultivation and the lag in genetic engineering development.

## Materials and methods

### Database construction

MethArDB was developed by integrating publicly available genomic resources for methanogenic archaea from multiple authoritative repositories. Genome data were collected from the NCBI GenBank [29], the GTDB, and NCBI RefSeq [30, 31], which provide genome assemblies and taxonomic information for cultured isolates and MAGs. Information on plasmids was obtained from GenBank, PlasmidScope [32], PLSDB [33], IMG/PR, and DDBJ [34, 35]. Phage records associated with methanogenic archaea were compiled from GenBank, RefSeq, PhageScope, and PhagesDB [36, 37]. These resources complement one another and collectively provide genome sequences together with mobile genetic element information that cannot be obtained from a single repository. Representative screenshots illustrating the MethArDB web interface and database organization are provided (Supplementary Fig. S1).

Data collection was performed by retrieving publicly available records from the corresponding databases. Because the same genome may be indexed in more than one resource, accession numbers were used as the primary identifiers during data integration. Records referring to the same genome assembly were compared across databases to eliminate duplicate entries while preserving complementary annotation information whenever available. This procedure generated a non-redundant reference dataset for downstream database construction.

To improve the consistency of the integrated resource, metadata associated with each genome were extracted from the original repositories, including assembly accession, organism name, taxonomic classification, assembly level, isolation source, geographical origin, and associated plasmid or phage information when available. Taxonomic classifications in MethArDB follow those provided by the original source databases, primarily GTDB and NCBI Taxonomy. Genome assembly status is presented using the official Assembly Level definitions provided by NCBI (Complete Genome, Scaffold, and Draft) [38], allowing users to distinguish genome assemblies with different assembly statuses.

The current release of MethArDB contains 3919 methanogenic archaeal genomes together with the associated plasmid and phage records collected from the public databases described above. To improve data accessibility and ensure long-term availability, all integrated datasets have been permanently archived in the Zenodo repository (DOI: 10.5281/zenodo.20528636), where each database release is preserved as a versioned record. Users can browse and analyse the data through the MethArDB web interface or download the complete datasets directly from Zenodo for offline use. The complete list of genomes included in the current release, together with their accession numbers, assembly levels, source databases, and associated metadata, is provided in the supplementary material (Supplementary Data S1).

### Genome optimization tool

Although MethArDB integrates publicly available methanogenic archaeal genomes, a substantial proportion of them are currently represented by draft assemblies. To facilitate downstream analyses using incomplete genome assemblies, a genome optimization workflow was incorporated into the MethArCT analysis pipeline. The workflow consists of four consecutive steps, including genome optimization, genome quality assessment, genome annotation, and MethArCT analysis (Supplementary Fig. S2).

In the genome optimization step, user-uploaded draft genome assemblies in FASTA format are first processed using RagTag (v2.1.0) [11], which performs reference-guided scaffolding by aligning contigs to a closely related reference genome and extending scaffold continuity. The resulting scaffolds are subsequently processed using Ragout (v2.3.0) [8], which reconstructs chromosome-scale scaffold order based on conserved synteny among reference genomes. Together, these two tools improve genome continuity and generate scaffold assemblies suitable for downstream analyses.

The optimized genome assemblies are subsequently evaluated using CheckM2 (v1.1.0) [13], which estimates genome completeness and contamination. These quality metrics allow users to assess assembly reliability before functional annotation.

Genome annotation is performed using Bakta (v1.11.4) [39], which predicts protein-coding genes and generates standardized protein sequence files (.faa). The resulting protein sequences serve as the input for subsequent MethArCT analyses.

Based on the annotated protein sequences generated by Bakta, MethArCT performs downstream analyses, including methanogenesis pathway prediction, optimal growth temperature prediction, salinity tolerance prediction, and culturability prediction. EzAAI (v1.2.4) [10] is integrated for average amino acid identity calculation and genome similarity analysis, while phylogenetic trees are constructed to support comparative genomic analyses.

The genome optimization workflow integrates multiple bioinformatics tools within the MethArCT platform, allowing users to improve draft genome assemblies, evaluate genome quality, perform genome annotation, and conduct downstream comparative analyses through a unified web interface (Table 1).

**Table 1:** Bioinformatics analysis tools integrated into MethArDB.

Name	Role	Citation
RagTag (v2.1.0)	Reference-guided scaffolding of draft genome assemblies	[11]
Ragout (v2.3.0)	Reference-assisted chromosome reconstruction and scaffold ordering	[8]
CheckM2 (v1.1.0)	Genome quality assessment through completeness and contamination estimation	[13]
Bakta (v1.11.4)	Genome annotation and protein-coding gene prediction	[39]
EzAAI (v1.2.4)	Average amino acid identity calculation and genome similarity analysis	[10]

### MethArCT: a culturomics analysis toolkit

Methanogenic archaea are difficult to cultivate because of their strict anaerobic requirements, slow growth rates, and frequently low abundance in environmental samples. To facilitate genome-based prediction of physiological traits, we developed MethArCT, a culturomics analysis toolkit integrated into the MethArDB platform. MethArCT performs downstream analyses using protein sequences generated by Bakta annotation or protein sequences directly submitted by users. The protein sequences are compared against a manually curated marker gene dataset specifically developed for methanogenic archaea (Supplementary Data S2). The resulting marker gene presence/absence profiles are subsequently used to infer metabolic pathways, optimal growth temperature, salinity tolerance, and predicted culturability. The workflow comprises the following analytical steps:

- *Input*. Protein sequence files (.faa) generated by Bakta annotation or provided directly by users.
- *Analysis of substrate metabolism*. Marker genes involved in sulphur metabolism, nitrogen metabolism, and methanogenesis are identified by sequence similarity searches against the reference gene dataset. The metabolic analyses include sulphur metabolism, nitrogen metabolism, and methanogenesis, with methanogenesis being represented by the most comprehensive collection of functional marker genes. The methanogenesis module covers formate, carbon dioxide, methoxy groups, methanol, methanethiol, methylamine, dimethylamine, trimethylamine, fatty acids, acetate, carbon monoxide, and long-chain alkanes. These analyses provide predictions of potential carbon substrates for cultivation. The complete list of marker genes used for pathway prediction is provided in the supplementary material (Supplementary Data S2).
- *Analysis of optimal growth temperature*. MethArCT utilizes TOME [40] to analyse input protein sequences and predict the optimal growth temperature of the target organism. The predicted temperature is subsequently classified into corresponding physiological temperature categories for cultivation guidance.
- *Analysis of culturability*. Culturability is predicted based on the distribution and relative abundance of manually curated marker genes associated with biosynthetic capacity, energy conservation, environmental adaptation, and genetic information processing. According to the proportion of detected functional marker genes, MethArCT assigns genomes to different predicted cultivation categories. The predicted cultivation categories include parasitic, symbiotic, and independently cultivable microorganisms.
- *Analysis of salinity tolerance*. Salinity tolerance is inferred from the distribution of genes involved in osmotic adaptation, ion transport, and compatible solute metabolism, allowing prediction of salt tolerance levels for cultivation.
- *Output*. It includes a genome quality chart, a comprehensive cultivation assessment chart, a methanogenic substrate type chart, and a TSV summary table reporting substrate metabolism capabilities, predicted growth temperature, cultivation mode, and salinity tolerance.

### Dual-threshold alignment strategy

A dual-threshold alignment strategy was employed, where the E-value for most orthologous proteins was set to <1e^−100^ based on analysis, whereas the threshold for paralogous proteins was set to E-value <1e^−5^ [41]. These thresholds were empirically determined based on benchmarking against experimentally validated gene sets (Supplementary Data S3), enabling effective discrimination between closely related orthologous and paralogous proteins.

## Results

### Overview of the MethArDB platform

We introduce MethArDB (http://methardb.cn), a comprehensive database dedicated to methanogenic archaea that addresses two major challenges in the field: fragmented genomic resources and the scarcity of functional annotations for genetic manipulation elements in plasmids. The database is structured into three core modules: genome, plasmid, and phage. This resource provides 3919 genomes, 87 plasmids, and 42 archaeal phage sequences sourced from all currently available public repositories (Fig. 1). This collection spans diverse taxonomic groups and environmental sources represented in currently available public databases (Supplementary Fig. S3). A key feature is the separate categorization of complete genomes, which enables direct access and analysis without preprocessing and thereby improves accessibility and streamlines downstream workflows. To ensure long-term preservation and reproducibility, all curated datasets included in MethArDB have been deposited in the Zenodo repository (DOI: 10.5281/zenodo.20528636). As shown in Fig. 1, the platform incorporates MethArCT, which leverages protein sequence analysis to predict key physiological traits (e.g. substrate specificity, temperature and salinity optima, and general culturability) and suggests potential cultivation strategies based on these predictions. MethArDB also provides integrated genomics tools including RagTag and Ragout for assembly improvement, CheckM2 for quality evaluation, and additional utilities for comparative analysis.

### Genome, plasmid, and phage database modules

**Figure 2:**
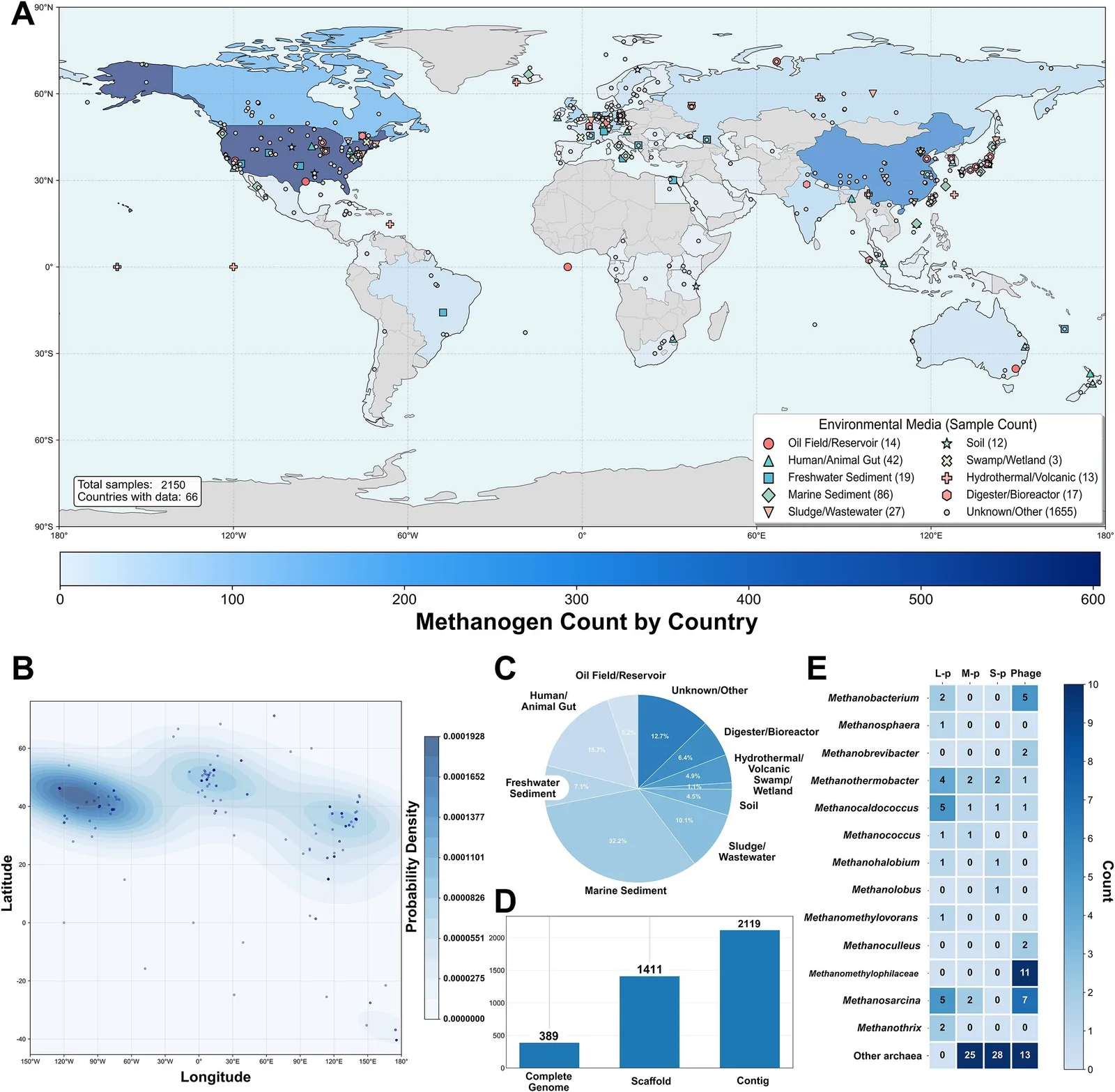
The genome, plasmid, and phage resources in MethArDB. (A) Geographic distribution of environmental sources for 2150 methanogenic archaea genomes. (B) Kernel density estimation (KDE) plot of the geographic distribution of complete methanogenic archaeal genomes. (C) Pie chart showing the environmental sources of complete methanogenic archaeal genomes. (D) Summary of genomes included in MethArDB. (E) Summary of plasmids and phages included in MethArDB.

Genome sequences provide the foundation for studying the taxonomy, evolution, and metabolic diversity of methanogenic archaea. MethArDB currently curates 3919 methanogenic archaeal genomes, including 363 complete genomes and 1411 scaffold-level assemblies (Fig. 2D). Geographic metadata are available for ∼2150 genomes (Supplementary Data S4), which form the basis for the visualization of their global distribution (Fig. 2A). Overall, roughly half of all archived genomes were sourced from the RefSeq and GenBank databases. The taxonomic profile of the database at both phylum and genus levels is summarized (Supplementary Fig. S3). The current dataset is dominated by genomes recovered from intestinal and marine sediment environments, whereas genomes from many other habitats remain relatively limited.

Plasmids and archaeal phages represent foundational tools in the life sciences, playing indispensable roles in genetic engineering, synthetic biology, and metabolic engineering research. Among these, plasmids are widely used as standardized vectors for gene cloning and expression, providing a fundamental platform for genetic manipulation and metabolic pathway reconstruction. Archaeal phages, with their efficient gene delivery and precise genome-editing capabilities, have emerged as critical tools for functional exploration and system redesign. Together, these two tools provide powerful resources for deciphering biological mechanisms and constructing artificial biological systems, laying a solid foundation for applications ranging from basic research to industrial biomanufacturing.

In this context, research on plasmids of methanogenic archaea is of considerable scientific interest and may contribute to advancing carbon neutrality and the development of bio-methane energy technologies [42]. Our database systematically catalogues 87 publicly available methanogenic archaeal plasmids (Fig. 2E). These plasmid sequences provide valuable resources for comparative analyses of archaeal plasmids and future studies of archaeal genetic systems. The database also includes 42 archaeal phage genomes (Fig. 2E), providing resources for studies of phage-derived functional proteins, archaeal antiviral defence systems, genome editing strategies, and phage-mediated gene transfer.

### Genome optimization and analysis workflow in MethArDB

MethArDB integrates RagTag, Ragout, CheckM2, Bakta, and EzAAI into a web-based genome optimization workflow, allowing users to perform genome assembly improvement, quality assessment, annotation, and comparative analyses through a graphical interface without requiring command-line expertise.

**Figure 3:**
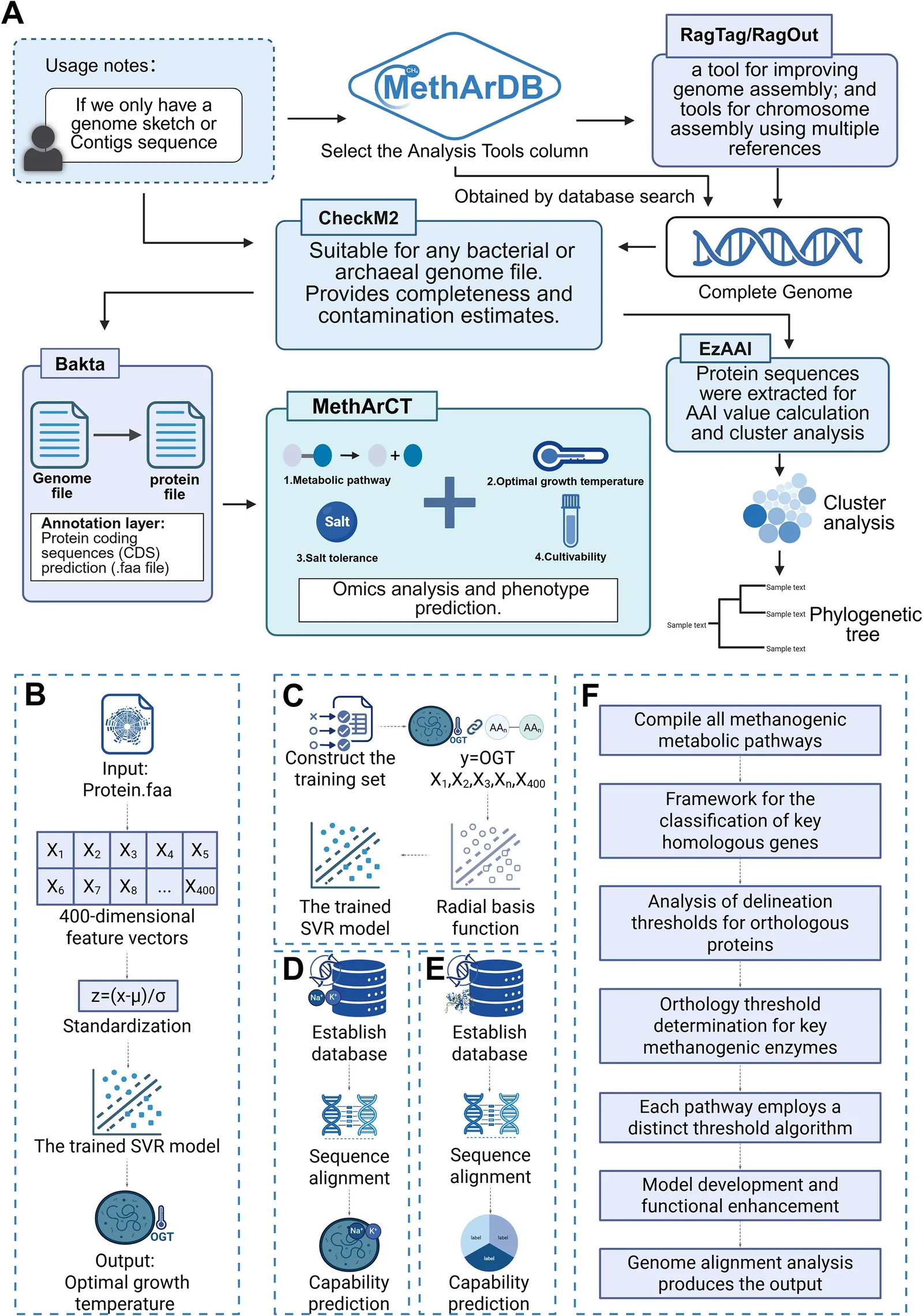
Principles of the MethArCT bioinformatics tools integrated into MethArDB. (A) Overview and analysis pipeline of the integrated bioinformatics package in MethArDB. (B) Mechanism of the temperature prediction module in MethArCT. (C) Machine learning training framework for the temperature prediction module in MethArCT. (D) Workflow for salinity prediction in MethArCT. (E) Workflow underlying MethArCT’s culturability predictions. (F) Workflow for substrate metabolism prediction in MethArCT.

Specifically, RagTag performs reference-guided scaffolding of draft genome assemblies, whereas Ragout further reconstructs scaffold order to improve assembly continuity (Fig. 3A). Genomes optimized through this workflow can then be evaluated for completeness and reliability using CheckM2, providing guidance for users to select high-quality sequences. After obtaining high-quality genomes, users can subsequently annotate optimized genomes with Bakta and perform average amino acid identity analysis using EzAAI to support comparative genomic and phylogenetic analyses.

Overall, the integrated workflow enables users to process draft methanogenic archaeal genomes through assembly improvement, quality assessment, genome annotation, and comparative genomic analyses within a unified web platform. A detailed schematic of the genome optimization workflow is provided (Supplementary Fig. S2).

### MethArCT: a culturomics toolkit integrated into MethArDB

In addition to integrating third-party genome optimization tools, we independently developed and incorporated MethArCT, a genome analysis toolkit that was designed specifically for archaea, particularly methanogenic archaeal groups. This toolkit is implemented as a Python package. It integrates a manually curated marker gene dataset together with established bioinformatics methods to infer cultivation characteristics of methanogenic archaea. Using protein sequences generated by Bakta annotation or provided directly by users, MethArCT predicts key physiological traits relevant to cultivation, including potential growth temperature ranges (Fig. 3B), salinity tolerance (Fig. 3D), predicted cultivation mode (Fig. 3E), and metabolically available substrates (Fig. 3F). These predictions provide guidance for archaeal isolation and cultivation strategies.

**Figure 4:**
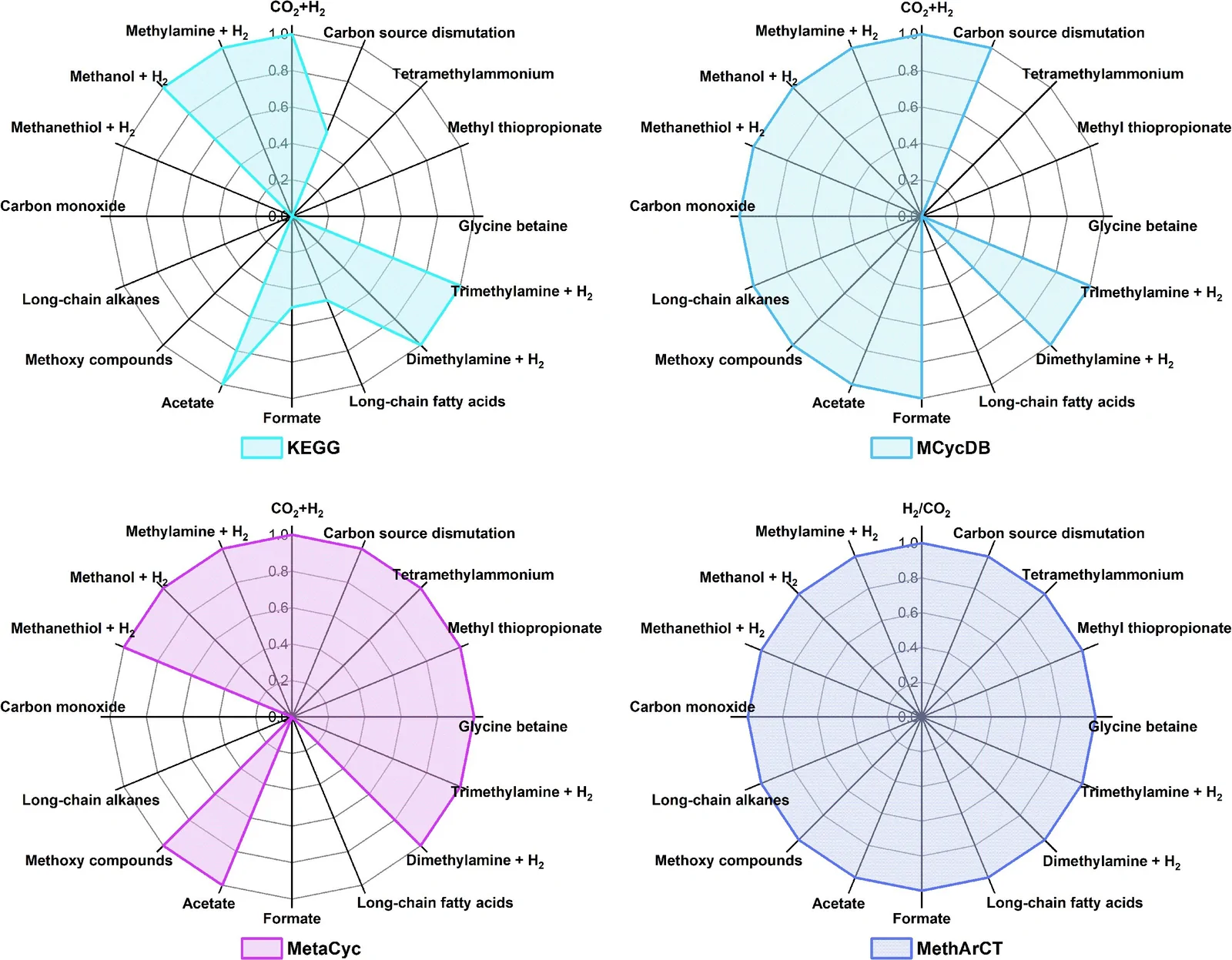
Comparison of methanogenesis pathway coverage among KEGG, MCycDB, MetaCyc, and MethArCT. Radar plots summarize the coverage of the 16 methanogenesis pathways evaluated in this study. Coverage was determined by manually examining whether each database explicitly contains the representative marker genes used to identify each methanogenesis pathway (Supplementary Data S2). Pathway names shown around the radar plots correspond to the substrate categories summarized in Table 2, whereas the representative marker genes and detailed database coverage are provided in Table 2. MethArCT incorporates marker genes for all currently reported methanogenesis pathways included in this comparison.

MethArCT integrates 16 experimentally reported methanogenesis pathways, including CO₂/H₂, formate, methanol (methyl-reducing), methylamine, dimethylamine, trimethylamine, methanethiol, methanol (carbon source dismutation methanogenesis), acetate, carbon monoxide, methoxy compounds, long-chain alkanes, long-chain fatty acids, tetramethylammonium, glycine betaine, and methylthiopropionate (Table 2; Supplementary Data S2) [15, 16, 20, 22, 43–51]. Compared with KEGG, MCycDB, and MetaCyc, MethArCT provides more comprehensive coverage of experimentally reported methanogenesis pathways. Figure 4 summarizes pathway coverage across databases, whereas Table 2 lists the representative marker genes used for pathway identification. As a widely used metabolic pathway database, the KEGG includes five major categories and three derived branches of methanogenic metabolic pathways (Fig. 4). However, database updates are often delayed, resulting in limited coverage of novel methanogenic pathways reported in recent years [7]. For instance, KEGG provides limited coverage of certain recently reported methanogenic pathways and may not fully capture all key enzymes involved in specific reactions (Table 2).

**Table 2:** Comparison of methanogenesis pathway coverage and representative marker genes across databases.

Substrate type	Pathway type	Representative marker genes	Kegg	MetaCyc	MCycDB	MethArDB
Carbon dioxide and hydrogen	CO_2_-reducing methanogenesis	FwdA-H, Mvh/Elp	√	√	√	√
Formate	CO_2_-reducing methanogenesis	FdhAB	°	×	√	√
Methylamine and hydrogen	Methyl-reducing methanogenesis	MtbABC	√	√	√	√
Dimethylamine and hydrogen	Methyl-reducing methanogenesis	MtmBC	√	√	√	√
Trimethylamine and hydrogen	Methyl-reducing methanogenesis	MttBC	√	√	√	√
Methanol and hydrogen	Methyl-reducing methanogenesis	MtaABC	√	√	√	√
Methanethiol and hydrogen	Methyl-reducing methanogenesis	MtsA_1_/A_2_	×	√	√	√
Methanol	Carbon source dismutation methanogenesis	MtaABC	°	√	√	√
Acetate	Carbon source dismutation methanogenesis	Acs or Ack/Pta	√	√	√	√
Carbon monoxide	Carbon source dismutation methanogenesis	AcsBCD	×	√	√	√
Long-chain alkanes	Carbon source dismutation methanogenesis	AcrABG	×	×	√	√
Long-chain fatty acids	Carbon source dismutation methanogenesis	FadD, AcsL	°	×	×	√
Methoxy compounds	Carbon source dismutation methanogenesis	MtoABCD	×	×	√	√
Tetramethylammonium	Carbon source dismutation methanogenesis	MtqABC	×	√	×	√
Glycine betaine	Carbon source dismutation methanogenesis	MtgB	×	√	×	√
Methyl thiopropionate	Carbon source dismutation methanogenesis	MtpA	×	√	×	√

Note: ‘√’ indicates the pathway is represented with all key marker genes; ‘°’ indicates partial representation; ‘×’ indicates the pathway is absent or not represented.

The Methane Cycling Database (MCycDB) is a specialized database for methane cycling in environmental microbiomes (Fig. 4) and contains marker genes for 12 methanogenesis pathways [12]. However, it does not currently include marker genes associated with methanogenesis from long-chain alkanes, methoxy compounds, or long-chain fatty acids. MetaCyc is a comprehensive metabolic database covering all domains of life (Fig. 4) [52]. Although it contains extensive information on metabolic pathways and enzymes, it is not specifically designed for methanogenic archaea and therefore lacks several recently described methanogenesis pathways and their associated marker genes (Table 2).

By contrast, the methanogenesis analysis module implemented in MethArCT incorporates marker genes for all methanogenesis pathways currently reported in the literature (Fig. 4), providing comprehensive coverage for methanogen-specific pathway analysis. In addition to well-established pathways, it also includes recently described pathways associated with long-chain alkanes and long-chain fatty acids, thereby extending pathway coverage beyond currently available public metabolic databases.

### Dual-threshold alignment strategy in MethArCT

The occurrence of false positives in bioinformatics is a major reason for the continued reliance on wet-lab experiments and remains a long-standing challenge in this field. Although our understanding of methanogenic metabolic pathways has become increasingly systematic, accurately predicting the metabolic functions of methanogenic archaea remains a substantial challenge in bioinformatics.

This limitation stems mainly from the overreliance of traditional gene annotation methods on sequence similarity. In methanogenic archaea, numerous key protein families contain large numbers of paralogous proteins. These proteins exhibit high similarity in their sequence and three-dimensional structure, yet they possess distinct functional specificities. Examples include the methyltransferase family: MtsA (methanethiol methyltransferase), MtbA (methylamine methyltransferase), and MtaA (methanol methyltransferase); the iron hydrogenase family: FdhAB (formate dehydrogenase) and ElpAB (hydrogenase); the methyl-coenzyme M reductase family: AcrABG (alkane-coenzyme M reductase) and McrABG (methyl-coenzyme M reductase). Annotation of such functionally distinct paralogous proteins based solely on sequence similarity can lead to functional misclassification, thereby undermining the reliability of metabolic pathway reconstruction.

To reduce false-positive annotations during metabolic pathway reconstruction, MethArCT implements a dual-threshold alignment strategy. The method first identifies homologous proteins by sequence similarity and then applies different E-value thresholds to distinguish orthologous proteins from closely related paralogous proteins. This strategy improves the accuracy of functional annotation and consequently increases the reliability of methanogenesis pathway prediction.

**Figure 5:**
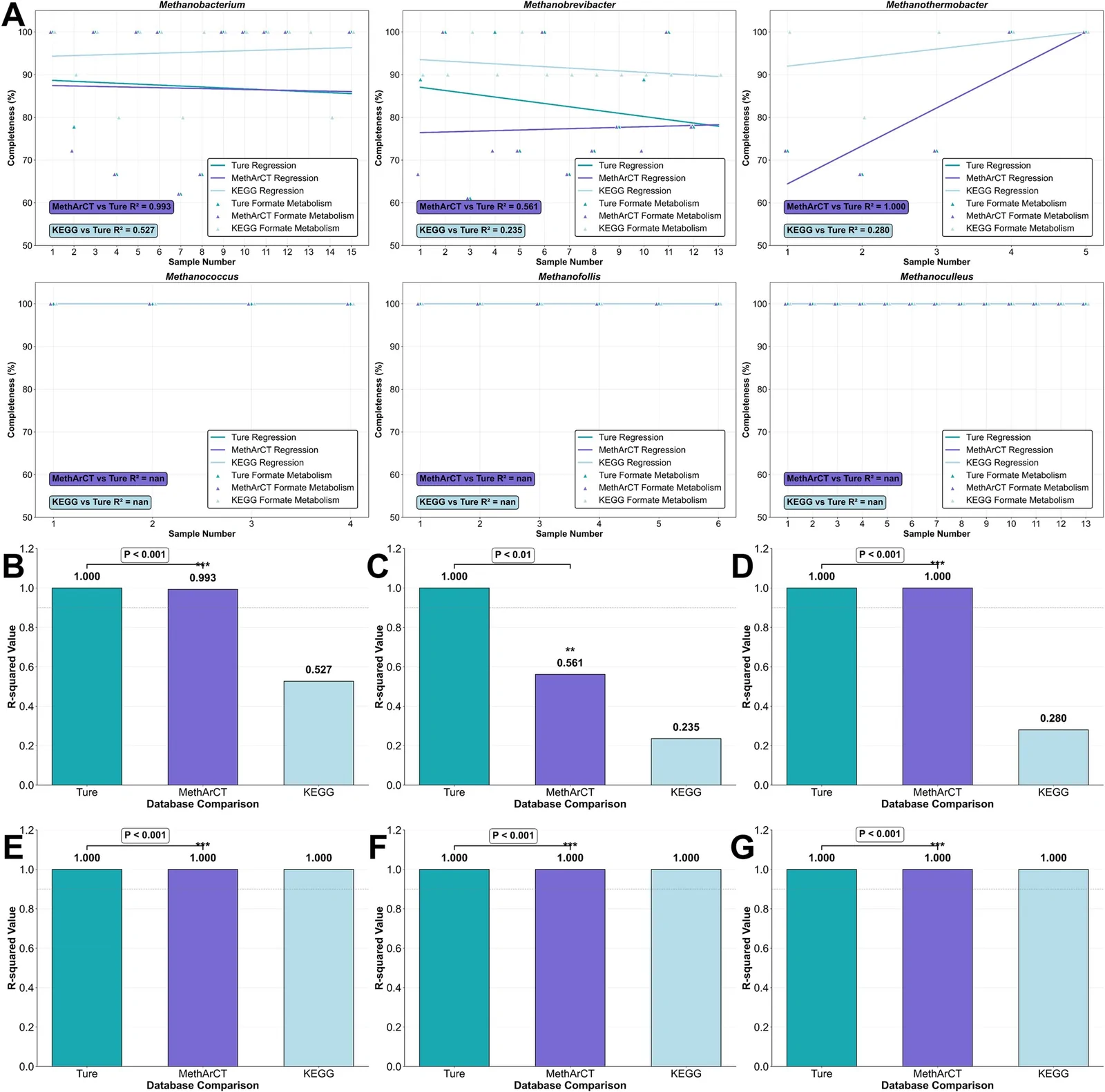
Evaluation of MethArCT using the dual-threshold alignment strategy. (A) Accuracy comparison between MethArCT and KEGG in predicting formate metabolism across six methanogenic groups. (B) Accuracy comparison between MethArCT and KEGG in predicting the formate methanogenesis pathway in *Methanobacterium*. (C) Accuracy comparison between MethArCT and KEGG in predicting formate-derived methanogenesis of *Methanobrevibacter*. (D) Accuracy comparison between MethArCT and KEGG in predicting formate-derived methanogenesis of *Methanothermobacter*. (E) Accuracy comparison between MethArCT and KEGG in predicting formate-derived methanogenesis of *Methanococcus*. (F) Accuracy comparison between MethArCT and KEGG in predicting formate-derived methanogenesis of *Methanofollis*. (G) Accuracy comparison between MethArCT and KEGG in predicting formate-derived methanogenesis of *Methanoculleus*.

To evaluate the performance of the dual-threshold alignment strategy, we analysed representative type species from six methanogenic genera (*Methanobacterium, Methanobrevibacter, Methanothermobacter, Methanococcus, Methanofollis*, and *Methanoculleus*) retrieved from the NCBI database. *Methanobacterium* is generally recognized as an obligate hydrogenotrophic methanogenic microbial group; however, some species have been reported to possess the capacity for concurrent formate metabolism. This likely results from the fact that ElpA and ElpB, two subunits of the iron hydrogenase complex ElpABC-HdrABC (which acquires reducing power from hydrogen), are homologous to FdhA and FdhB, the two subunits of formate dehydrogenase (Fig. 5A). However, BlastKOALA, KEGG’s metabolic pathway analysis tool, often fails to account for this homology, resulting in a high false-positive rate. Consequently, the *R*^2^ value between predictions and the true values was 0.527, indicating a relatively low level of agreement. The true values were defined based on experimentally validated metabolic capabilities reported in the literature for representative genera, compiled from published studies [46]. In contrast, MethArCT applies more stringent alignment thresholds for several key homologous enzymes involved in the hydrogenotrophic methanogenic pathway (CO₂-reduction pathway) and the formate-based methanogenic pathway to distinguish between paralogous and orthologous proteins (Supplementary Data S3). This improves prediction accuracy and reliability, with an *R*^2^ value of 0.993 between MethArCT’s predictions and the true values. Similar improvements were observed for *Methanothermobacter* and *Methanobrevibacter*, two genera that also rely primarily on hydrogenotrophic methanogenesis. For both groups, MethArCT produced pathway predictions that more closely matched experimentally validated metabolic phenotypes than those generated using KEGG (Fig. 5B and C). *Methanoculleus* utilizes both hydrogen and formate for methanogenesis [53–56]. MethArCT correctly identified both pathways, demonstrating that the dual-threshold strategy is applicable to methanogens with more diverse metabolic capabilities (Fig. 5F).

This study aimed to evaluate the performance of the MethArCT analysis package in predicting the completeness of metabolic pathways in methanogens. We compared results from MethArCT and the widely used KEGG database with the true values to assess the completeness of CO₂ reduction and formate metabolism pathways across six major methanogen genera (*Methanobacterium, Methanothermobacter, Methanobrevibacter, Methanococcus, Methanofollis*, and *Methanoculleus*). Although false positives are virtually unavoidable in bioinformatics analysis, MethArCT exhibited high consistency with true values when predicting pathway completeness across all studied methanogen genera (mean Pearson’s *R*² of MethArCT > 0.8, and Pearson’s *R*² of MethArCT ≫ Pearson’s *R*² of KEGG). These results demonstrate that the dual-threshold alignment strategy implemented in MethArCT improves the accuracy of metabolic pathway predictions in methanogens, thereby providing a reliable tool for advancing our understanding of the methane biogeochemical cycle.

## Discussion

This study describes the MethArDB database and its integrated analysis toolkit, MethArCT, which aims to address long-standing challenges in methanogenic archaea research, including difficulties in cultivation and isolation, insufficient understanding of genetic mechanisms as reflected by incomplete functional annotation and a large number of genes with unknown functions, and limited genetic engineering technologies. This platform integrates high-quality genomic and mobile genetic element resources that may facilitate future genetic engineering studies of methanogenic archaea. It facilitates the analysis of methanogenic potential and metabolic pathways through high-precision metabolic pathway prediction and provides reliable predictions of key physiological traits (e.g. optimal temperature, salinity tolerance, culturability), thereby supporting the development of strategies for the isolation and cultivation of difficult-to-culture strains. Overall, the establishment of MethArDB/MethArCT provides a foundation for in-depth analysis of methanogenic pathways, advancement of genetic engineering applications, and development of methane-based bioenergy.

The MethArCT module employs a dual-threshold alignment strategy to distinguish orthologs from paralogs, reducing annotation errors caused by high sequence and structural similarity. This strategy improves the accuracy of metabolic pathway predictions, particularly for paralogous protein families that are structurally similar yet functionally distinct, such as MtaA and MtsA, or McrA and AcrA. Additionally, MethArCT compiles a comprehensive set of methanogenesis pathways, supporting a more comprehensive assessment of methanogenic potential and substrate diversity.

Moreover, MethArCT can predict key physiological traits of methanogenic archaea, including optimal growth temperature, salt tolerance, and culturability. Validation against experimental data from cultured strains in DSMZ and *Bergey’s Manual of Systematic Bacteriology* (BMSAB) demonstrated the module’s high predictive accuracy, providing useful guidance for designing laboratory cultivation strategies for methanogens. This predictive capability addresses the current lack of dedicated tools for predicting cultivation conditions for methanogenic archaea and holds promise for advancing research on rare, extremophilic, and uncultured methanogenic lineages.

Nevertheless, this study has several limitations. The current applicability of MethArCT to extreme environmental conditions is constrained by the limited availability of experimentally characterized reference datasets. In the future, we plan to extend MethArCT into a systems biology platform for archaea by incorporating gene regulatory and metabolic network analyses. We also plan to integrate multi-omics datasets, including transcriptomics, proteomics, and metabolomics, together with additional computational approaches to further improve the functionality and predictive performance of MethArCT. Continued development of this platform may provide theoretical and technical support for synthetic biology applications, including the design of methane-producing strains, environmental remediation, and methane-based bioenergy production. To ensure the long-term sustainability of MethArDB, future releases will incorporate newly available genomes, high-quality MAGs, plasmids, archaeal phages, and experimentally validated functional information through a combination of automated data retrieval and manual curation. Updated versions of the database will be archived in Zenodo to ensure long-term accessibility and reproducibility.

Together, MethArDB and MethArCT form a comprehensive platform for research on methanogenic archaea. By systematically integrating genomic, plasmid, and archaeal phage resources together with curated functional annotations, the platform provides resources that may facilitate future studies on methanogen genetic engineering. The innovative dual-threshold strategy enables more accurate metabolic pathway analysis, thereby improving the identification of key methanogenesis marker genes, such as ElpAB and FdhAB. The physiological trait prediction module further provides useful guidance for the isolation and cultivation of difficult-to-culture methanogenic archaea. Overall, MethArDB/MethArCT provides an integrated resource for studying methanogen genomics, physiology, and metabolism, thereby facilitating future research in archaeal microbiology and biotechnology.

## Supplementary Material

baag055_Supplemental_Files

## Data Availability

All genomic data analysed in this study were obtained from publicly available repositories, including NCBI GenBank, RefSeq, GTDB, KEGG, and other databases described in the Methods section. The complete MethArDB dataset, including curated methanogenic archaeal genome metadata, plasmid records, archaeal phage records, accession lists, and associated datasets, has been permanently archived in Zenodo under the Creative Commons Attribution 4.0 International License (CC BY 4.0) and is available at https://doi.org/10.5281/zenodo.20528636. The online implementation of MethArDB is available at http://methardb.cn. Source code for MethArCT is publicly available at https://github.com/rsj99-dev/MethArCT. Source data for all figures are provided with this paper.
