# Supplementary material for: An integrated culturomic and genomic database and analysis platform for methanogenic archaea
Source: Database (Oxford). 2026 Sep 15;2026:baag055. doi: 10.1093/database/baag055 (PMC13576065; doi:10.1093/database/baag055)
Supplement: Supplementary file 1 [file baag055_supplemental_files.zip › Supplementary Fig. S2.pdf]

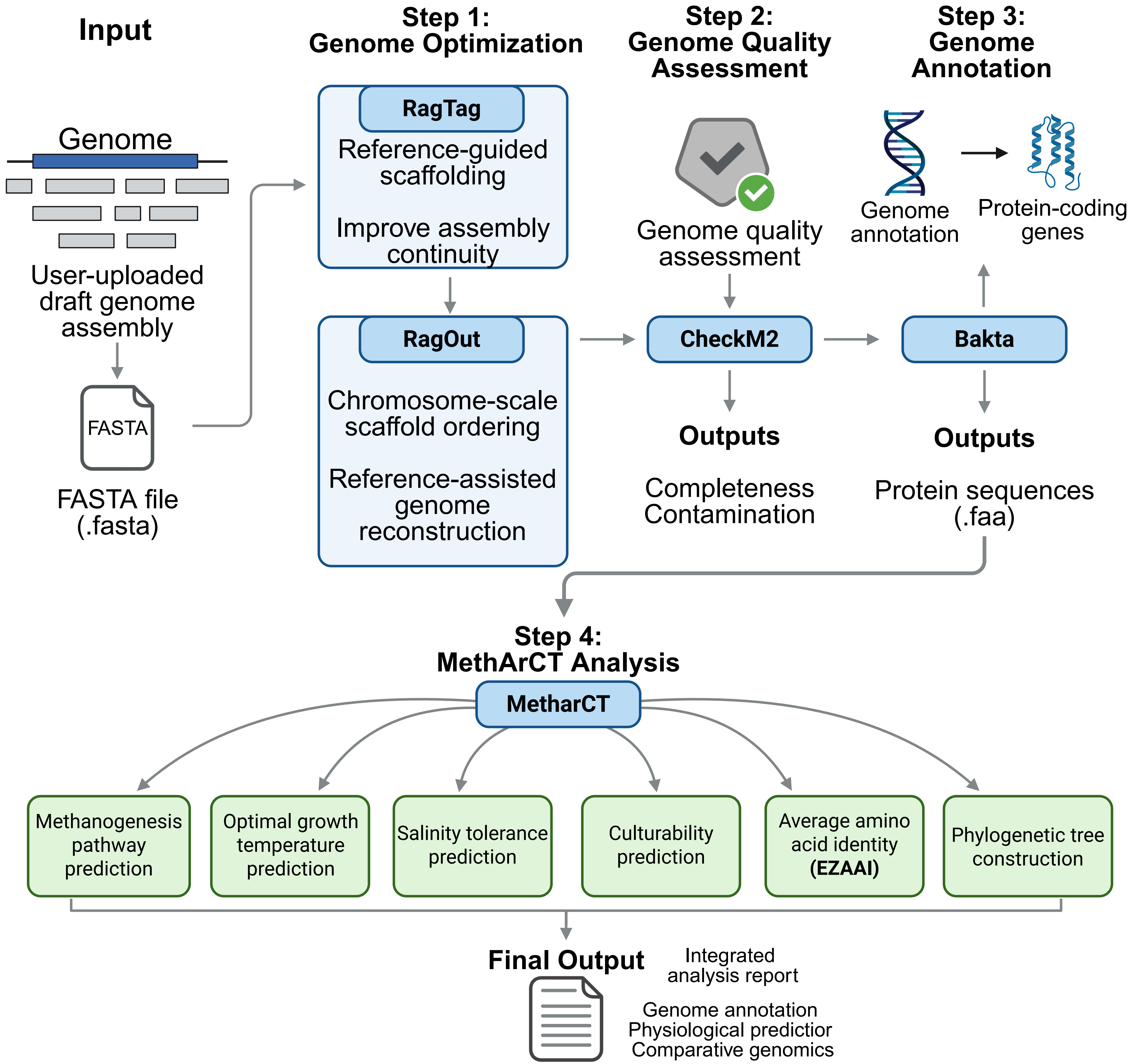

Input
Step 1:
Genome Optimization
Step 2:
Genome Quality Assessment
Step 3:
Genome Annotation
Genome
User-uploaded draft genome assembly
FASTA
FASTA file (.fasta)
RagTag
Reference-guided scaffolding
Improve assembly continuity
RagOut
Chromosome-scale scaffold ordering
Reference-assisted genome reconstruction
Genome quality assessment
CheckM2
Outputs
Completeness
Contamination
Genome annotation
Protein-coding genes
Bakta
Outputs
Protein sequences (.faa)
Step 4:
MethArCT Analysis
MetharCT
Methanogenesis pathway prediction
Optimal growth temperature prediction
Salinity tolerance prediction
Culturability prediction
Average amino acid identity (EZAAI)
Phylogenetic tree construction
Final Output
Integrated analysis report
Genome annotation
Physiological predictior
Comparative genomics
