# Supplementary material for: An integrated culturomic and genomic database and analysis platform for methanogenic archaea
Source: Database (Oxford). 2026 Sep 15;2026:baag055. doi: 10.1093/database/baag055 (PMC13576065; doi:10.1093/database/baag055)
Supplement: Supplementary file 1 [file baag055_supplemental_files.zip › Supplementary Fig. S3.pdf]

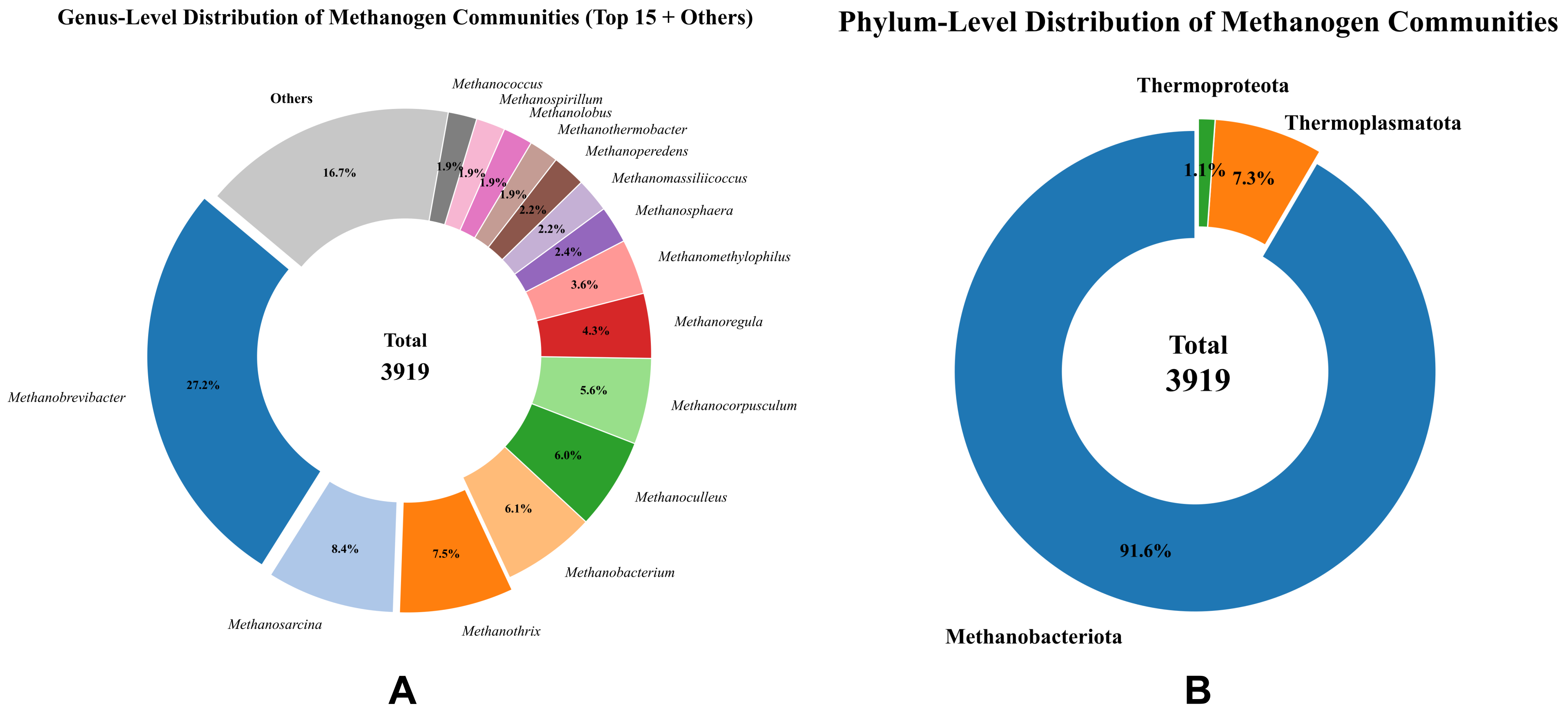
Genus-Level Distribution of Methanogen Communities (Top 15 + Others)
Others
16.7%
Methanococcus
1.9%
Methanospirillum
1.9%
Methanolobus
1.9%
Methanothermobacter
1.9%
Methanoperedens
2.2%
Methanomassiliicoccus
2.2%
Methanosphaera
2.4%
Methanomethylophilus
3.6%
Methanoregula
4.3%
Methanocorpusculum
5.6%
Methanoculleus
6.0%
Methanobacterium
6.1%
Methanothrix
7.5%
Methanosarcina
8.4%
Methanobrevibacter
27.2%
Total
3919
A
Phylum-Level Distribution of Methanogen Communities
Thermoproteota
1.1%
Thermoplasmatota
7.3%
Methanobacteriota
91.6%
Total
3919
B
