# Supplementary material for: An integrated culturomic and genomic database and analysis platform for methanogenic archaea
Source: Database (Oxford). 2026 Sep 15;2026:baag055. doi: 10.1093/database/baag055 (PMC13576065; doi:10.1093/database/baag055)
Supplement: Supplementary file 1 [file baag055_supplemental_files.zip › Supplementary Materials.pdf]

Supplementary Materials

Title:

An Integrated Culturomic and Genomic Database and Analysis Platform for Methanogenic Archaea

Authors:

Jiuyu Chen[1], Shijie Ren[1], Zhenye Tong[1], Zhaofeng Guan[1], Wenhao Zhang[1], Siwei Ren[1], Liqian Ma[1], Lingdou Kong[1], Hengyao Chong[1], Zihao Wang[1], Xiaoyu Yong[1], Su Yan[1], Yuanpeng Wang[2], Jun Zhou[1]*

Affiliations and addresses:

[1]Bioenergy Research Institute, College of Biotechnology and Pharmaceutical Engineering, Nanjing Tech University, Nanjing, Jiangsu 211816, China

[2]Department of Chemical and Biochemical Engineering, College of Chemistry and Chemical Engineering, Key Laboratory for Chemical Biology of Fujian Province, Xiamen University, Xiamen, Fujian 361005, China

*Corresponding author: Dr. Jun Zhou

Tel: +86 25 58139929

Fax: +86 25 58139929

E-mail address: zhoujun@njtech.edu.cn

Contents

Supplementary Figures

Supplementary Fig. S1. The MethArDB database website homepage and a list of genomic data.

Supplementary Fig. S2. Workflow of the genome optimization and analysis pipeline implemented in MethArCT.

Supplementary Fig. S3. Taxonomic composition of methanogenic archaea included in MethArDB.

Supplementary Data

Supplementary Data S1 Complete accession list of genomes, plasmids, and archaeal phages included in MethArDB

Supplementary Data S2 Marker gene datasets used by MethArCT for methanogenesis pathway identification, salinity tolerance prediction, and cultivation prediction

Supplementary Data S3 Benchmark dataset for evaluating the dual-threshold alignment strategy implemented in MethArCT

Supplementary Data S4 Geographic metadata used for global distribution analysis of methanogenic archaeal genomes

Additional files

High-resolution vector graphics (SVG) for Figure 2.

High-resolution vector graphics (SVG) for Figure 5.

Supplementary Figures

Fig. S1. The MethArDB database website homepage and a list of genomic data.

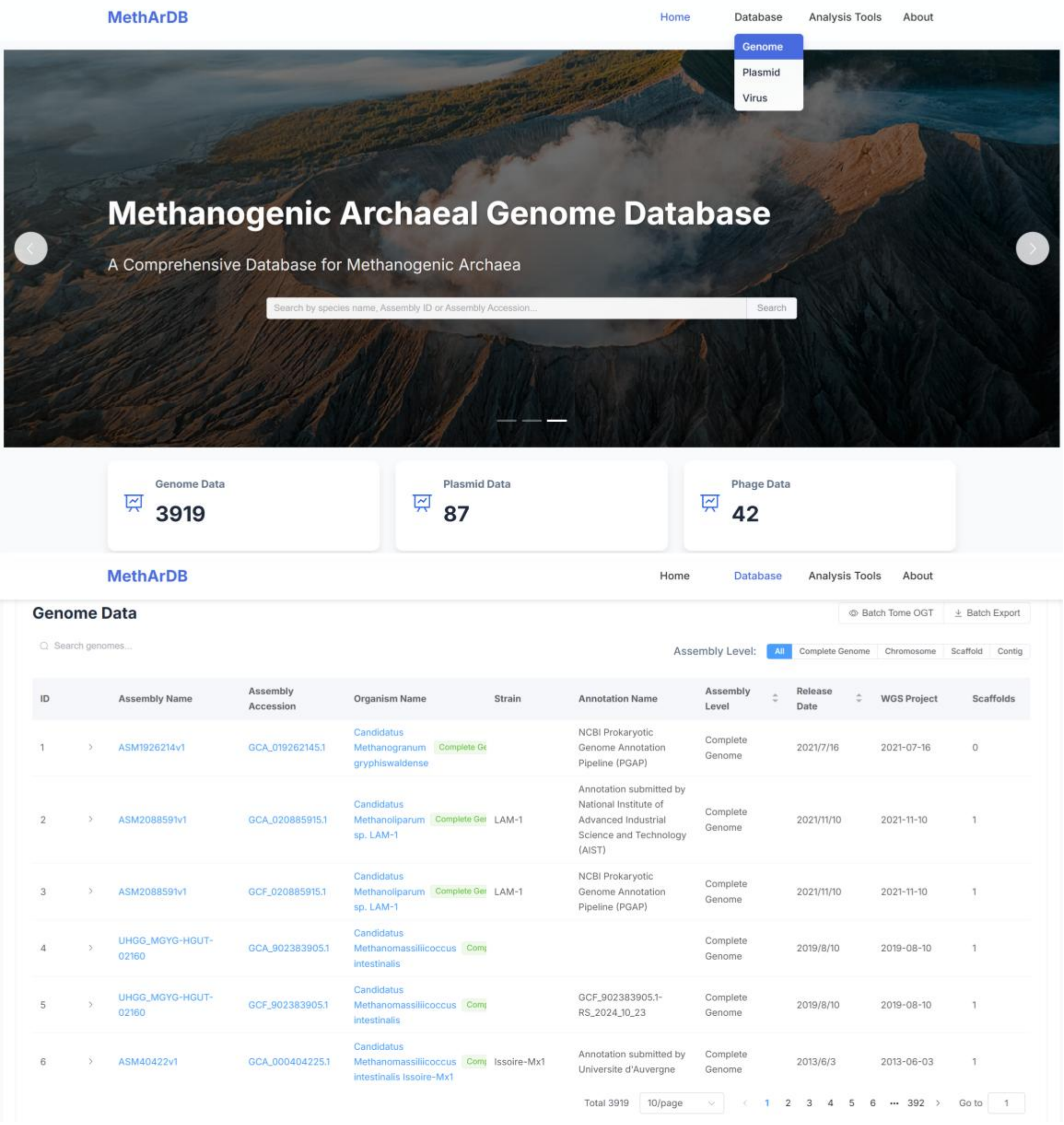

Homepage of the MethArDB website and overview of the database resources.

Fig. S2. Workflow of the genome optimization and analysis pipeline implemented in MethArCT.

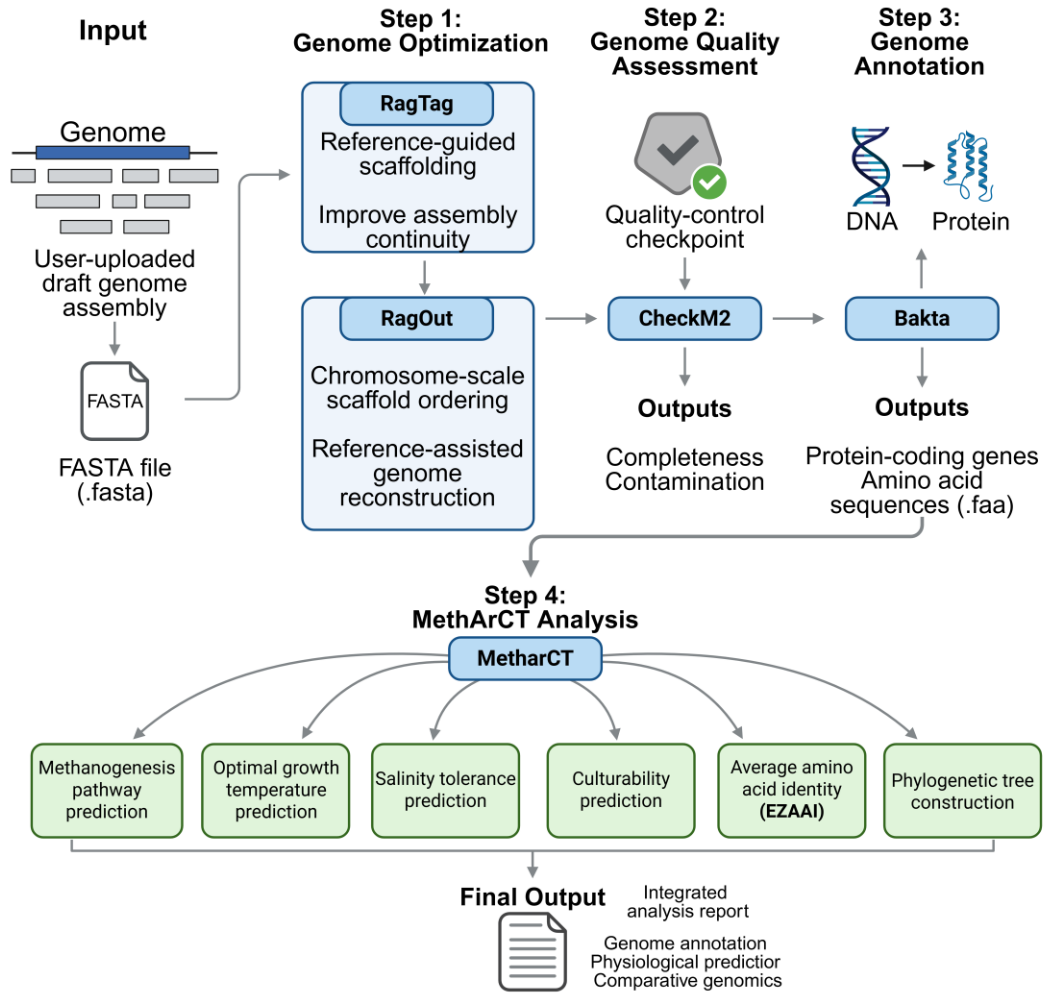

User-submitted draft genome assemblies are first optimized using RagTag and RagOut through reference-guided scaffolding and scaffold ordering. The optimized assemblies are subsequently evaluated with CheckM2 to estimate genome completeness and contamination. Protein-coding genes are predicted using Bakta, which generates amino acid sequence files (.faa) for downstream analyses. MethArCT uses the annotated protein sequences to perform methanogenesis pathway prediction, physiological trait prediction, average amino acid identity (AAI) analysis and phylogenetic reconstruction.

Fig. S3. Taxonomic composition of methanogenic archaea included in MethArDB.

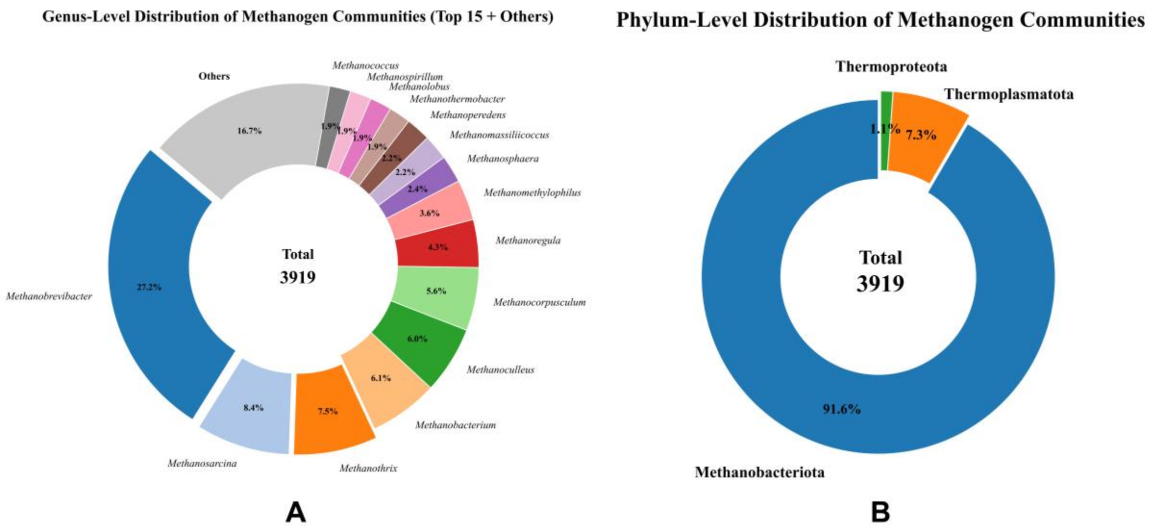

A. Distribution of curated methanogenic archaeal genomes at the genus level.

B. Distribution of curated methanogenic archaeal genomes at the phylum level.
